# Leaf Species-Dependent Fungicide Effects on the Function and Abundance of Associated Microbial Communities

**DOI:** 10.1007/s00128-023-03728-2

**Published:** 2023-05-09

**Authors:** Sara Gonçalves, Ruben Post, Marco Konschak, Jochen Zubrod, Alexander Feckler, Mirco Bundschuh

**Affiliations:** 1 iES Landau, RPTU Kaiserslautern-Landau, Fortstrasse 7, 76829 Landau, Germany; 2 Zubrod Environmental Data Science, Friesenstrasse 20, 76829 Landau, Germany; 3 Eußerthal Ecosystem Research Station, RPTU Kaiserslautern-Landau, Birkenthalstraße 13, 76857 Eußerthal, Germany; 4 Department of Aquatic Sciences and Assessment, Swedish University of Agricultural Sciences, Box 7050, 750 07 Uppsala, Sweden

**Keywords:** Recalcitrance level, Leaf traits, Aquatic fungi, Fungicides

## Abstract

Microbially-mediated leaf litter decomposition is a critical ecosystem function in running waters within forested areas, which can be affected by fungicides. However, fungicide effects on leaf litter decomposition have been investigated almost exclusively with black alder leaves, a leaf species with traits favourable to consumers (i.e., low recalcitrance and high nutrient content). At the same time, little is known about fungicide effects on microbial colonisation and decomposition of other leaf species with less favourable traits. In this 21 day lasting study, we explore the effects of increasing fungicide sum concentrations (0–3000 µg/L) on microbial colonisation and decomposition of three leaf species (black alder, Norway maple and European beech) differing in terms of recalcitrance and nutrient content. Leaf litter decomposition rate, leaf-associated fungal biomass and bacterial density were quantified to observe potential effects at the functional level. Beech, as the species with the least favourable leaf traits, showed a substantially lower decomposition rate (50%) in absence of fungicides than alder and maple. In the presence of high fungicide concentrations (300–3000 µg/L), beech showed a concentration-related decrease not only in microbial leaf litter decomposition but also fungal biomass. This suggests that favourable traits of leaf litter (as for alder and maple) enable leaf-associated microorganisms to acquire leaf-bound energy more easily to withstand potential effects induced by fungicide exposure. Our results indicate the need to deepen our understanding on how leaf species’ traits interact with the impact of chemical stressors on the leaf decomposition activity of microbial communities.

**Supplementary Information:**

The online version contains supplementary material available at 10.1007/s00128-023-03728-2.

Leaf litter decomposition is a key process in streams within forested catchments (Fisher and Likens 1973), which is *inter alia* driven by microbes such as bacteria and fungi, especially aquatic hyphomycetes (AH; Hieber and Gessner 2002). These microorganisms contribute directly to leaf litter decomposition, with their extracellular enzymes breaking down mono-, di- and polysaccharides (Evans and Hedger 2001). In this context, the efficiency of microorganisms to decompose leaf litter is assumed to be a function of microorganisms’ species-specific characteristics (Baudy et al. 2021) as well as the chemical composition of leaf species (Melillo et al. 1982; Hladyz et al. 2009; Schindler 2009). In fact, the levels of leaves’ nutrients and structural (recalcitrant) components influence microbial colonization dynamics (Melillo et al. 1982; Webster and Benfield 1986; Gessner and Chauvet 1994).

In addition, anthropogenic chemicals are known to alter microbial colonization and decomposition of leaf litter. One group of chemicals that received increasing attention over the last decade is fungicides, which are designed to affect fungal pest species in agriculture (Zubrod et al. 2019). After their application, fungicides can reach surface water bodies, for example via runoff (Süß et al. 2014), where they interact with non-target organisms, such as microorganisms involved in leaf litter decomposition (Zubrod et al. 2011; Feckler et al. 2017). However, most studies addressing fungicide effects on leaf litter decomposition used black alder (*Alnus glutinosa L.* (Gaertn.)) as a model leaf species (e.g., Bundschuh et al. 2011; Fernández et al. 2015). While black alder may be considered representative of temperate riparian ecosystems (Bjelke et al. 2016), leaf litter of other tree species is also ecologically highly relevant (Gessner et al. 2010). As black alder leaf litter has a high nutrient content paired with a low share of recalcitrant substances, such as lignin (e.g., Melillo et al. 1982; Gulis 2001), it becomes the first to be colonized and decomposed by microorganisms. At the same time, the decomposition of other leaf species with less favourable traits happens slower, enabling the constant input of nutrients all year long (Gessner et al. 2010). Thus, the transferability of results obtained with black alder to other leaf litter species with deviating characteristics may be questioned.

In order to investigate the impact of different leaf species on the function of leaf-associated microbial communities under fungicide exposure, the present study made use of three leaf species with distinct characteristics: black alder (referred to as alder), which due to its characteristics has a slightly and substantially higher decomposition rate compared to Norway maple (*Acer platanoides* L.; referred to as maple) and European beech (*Fagus sylvatica* L.; referred to as beech; Gessner and Chauvet 1994; Abelho 2001). These leaf species were colonized by aquatic microorganisms while being exposed to increasing concentrations of a fungicide mixture over 21 days. Leaf litter decomposition rates were quantified as a functional endpoint. Additionally, ergosterol content (as a proxy for fungal biomass) and bacterial density were measured to quantify microbial abundance. We expected (i) that alder and maple will be decomposed faster than beech in absence of fungicides, (ii) fungicides will negatively affect leaf-associated microorganisms’ function, independent of the leaf species and (iii) the magnitude of fungicide effects on microbial leaf litter decomposition increases with increasing level of recalcitrance. This hypothesis is derived from the dynamic energy budget theory (Kooijman 2000) suggesting an elevated investment of energy to obtain nutrients from the leaves, leaving less for other processes including detoxification.

## Materials and Methods

Leaf material was collected in the vicinity of Landau, Germany: alder leaves were collected in autumn 2017 (49°11′N; 8°′5′O), while beech leaves and maple leaves were collected in autumn 2016 and 2015 (49°12′N; 8°′6′O), respectively. All leaves were stored at − 20°C until use. To generate a near-natural inoculum of leaf-associated microorganisms, alder leaves were submerged in litterbags (mesh size: 0.5 mm; 10 leaves per bag) for 14 days in the Rodenbach, Germany (49°33′N, 8°′2′O). Subsequently, leaves were cleaned under tap water to remove adhering sediment and submerged for another 28 days in a stainless-steel channel filled with nutrient medium (Dang et al. 2005) being renewed every 7 days, under constant aeration and in darkness at 16 ± 1°C. Unconditioned alder leaves were added to generate an inoculum of various decomposition stages supposedly harbouring a higher fungal diversity (Gessner et al. 1993). This inoculum was subsequently used for the fungicide exposure assay.

For each leaf species, 150 unconditioned leaves were cut to strips (approximately 7.5 × 5 cm^2^). Leaf strips were leached for 24 h in nutrient medium to reduce potential impacts of leachates on microbially-driven leaf litter decomposition during the experiment (Gessner et al. 1999). Subsequently, leaf strips were dried at 60°C for 24 h and weighted to the nearest 0.01 mg. Each replicate consisted of three dried and pre-weighed leaf strips, leading to a total of 50 replicates per leaf species to be evenly split among five fungicide treatments (n = 10). The fungicide mixture used in the present study was composed of five fungicides covering a wide range of modes of action (Table S1). Fungicide test concentrations were chosen following earlier studies (e.g., Zubrod et al. 2015) using a spacing factor of ten: 0 (fungicide-free control), 3, 30, 300 and 3000 μg/L, with proper spiking being confirmed elsewhere (e.g., Zubrod et al., 2015).

For the experiment, a fully-crossed 3 × 5-factorial test design was used. Each of the three leaf species was exposed to the five fungicide concentrations, including a fungicide-free control. Before test initiation, dried leaf strips were rehydrated for 24 h in nutrient medium before being introduced into mesh bags (mesh size: 0.5 mm). Mesh bags prevented the three leaf strips from sticking together and ensuring the accessibility of the leaf material for microorganisms. Each replicate consisted of a 1 L glass beaker filled with 750 mL nutrient medium, 3 g microbial inoculum (wet weight; i.e., of pre-conditioned leaves), the three leaf strips as well as the fungicide mixture. Experiments were conducted at 16 ± 1°C under continuous aeration and in darkness. To avoid evaporation of nutrient medium, the beakers were covered with plastic foil, while the medium was renewed every seven days (including fungicide stocks). After 21 days, all leaf strips were removed from the test system and two leaf discs with a diameter of 16 mm were punched out of each leaf strip with a cork borer. One leaf disc from each leaf strip was used for leaf mass quantification and dried at 60°C for 24 h. The second leaf disc from each leaf strip was fixed in 2% formaldehyde solution (with 0.1% sodium pyrophosphate) and stored at 4°C for bacterial density analysis. The remaining material of the leaf strips was collected for leaf decomposition measurements as well as for ergosterol analysis and was stored at − 20°C until further use. To quantify the leaf decomposition, the leaf discs for mass correction and the remaining leaf strips were freeze-dried for 24 h and weighed to the nearest 0.01 mg.

The leaf-associated ergosterol was quantified as a proxy for fungal biomass according to Gessner (2005). After extraction in alkaline methanol, ergosterol was purified by solid-phase extraction (Sep-Pak Vac RC tC18 500 mg sorbent, Waters) and quantified by high-performance liquid chromatography (1200 Series, Agilent Technologies). The bacterial density was quantified following (Buesing 2005). Briefly, bacterial cells were detached from the leaf discs using an ultrasonic probe (Sonopuls HD 2070 with TT 13 probe, both Bandelin, Germany) and filtered over aluminium oxide membrane filters (pore size 0.2 μm, Whatman). Filters were subsequently stained with SYBR Green II (Molecular Probes, Eugene, OR, USA). Twenty digital images were taken for each replicate under an epifluorescence microscope (Axio Scope.A1, Carl Zeiss Micro Imaging). Bacterial cells were counted using Axio Vision Rel 4.8 (Carl Zeiss Micro Imaging) and normalised to leaf dry mass.

The microbial leaf decomposition rate *k* (d^−1^) was calculated following Benfield et al., 2007. Concentration–response models (including lognormal, log-logistic, Weibull, Cedergreen–Ritz–Streibig, and Michaelis–Menten models) were fitted separately for alder, beech and maple to assess the functional response to the five tested fungicide concentrations. The best-fitting models were selected based on visual judgment and Akaike’s information criterion (all models and their respective parameters are reported in Table S4). The data on leaf decomposition, fungal biomass and bacterial density were checked for normal distribution and heteroscedasticity via Shapiro–Wilk and Levene’s tests, respectively. Significant influences of the factors “fungicide treatment” and “leaf species” as well as their interaction were examined using rank-based two-way analyses of variance (ANOVA). For each leaf species, differences between control and individual fungicide treatments were checked with Wilcoxon rank sum tests followed by Bonferroni correction (Zar 2010). Moreover, we base our interpretation on both statistical significance and effect sizes, considering the criticism of null hypothesis significance testing (i.e., the difference between treatments (Newman 2009)). R version 4.2.1 for Windows (R Core Team 2022) was used for the execution of the statistical tests and the creation of figures. The graphical abstract was created in BioRender.com.

## Results and Discussion

**Fig. 1:**
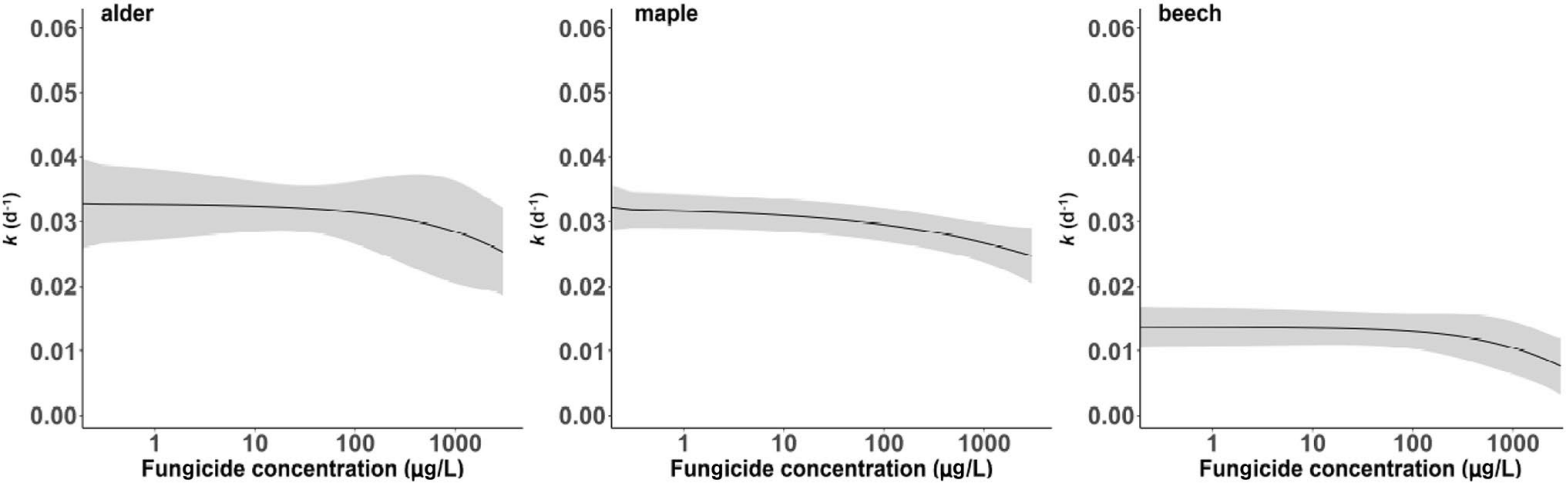
Concentration–response models (solid lines; shaded lines indicating corresponding 95% CIs; n = 10) for the leaf litter decomposition rate, *k* (d ^− 1^), as a function of the total fungicide concentration for the different leaf species alder, maple and beech

Leaf species significantly influenced the decomposition rate, fungal biomass and bacterial density (Fig. 1; Tables 1 and S3; *p* < 0.001). As hypothesised, beech leaves were decomposed slower than alder and maple in absence of fungicides. In general, alder leaves were decomposed fastest, followed by maple and beech (Fig. 1). This observation is in accordance with former studies (e.g., Abelho 2001) and is likely explained by a higher content of recalcitrant substances, such as lignin, in combination with low levels of nutrients in beech leaves (Melillo et al. 1982; Bastias et al. 2018). These leaf characteristics should restrict the colonisation of beech leaves by microbes, which in turn slows down decomposition. In contrast, leaf litter characterised by a lower recalcitrance and an elevated nutrient content (mainly nitrogen; Gulis 2001), such as maple and alder, should also support fungal growth and consequently being more efficiently degraded (Artigas et al. 2004; Graça and Canhoto 2006).

**Table 1:** Output for statistical analysis of the rank-based ANOVA

Enpoint	Method	Source of variation	Df	Sum Sq	Mean Sq	F value	*p*-value
Leaf litter decomposition rate	ANOVA	**Leaf species**	2	0.0107	0.0054	66.394	***p* < 0.001**
		**Fungicide**	4	0.0009	0.0002	2.824	**0.027**
		Leaf species × fungicide	8	0.0002	0.0001	0.387	0.926
		Residuals	135	0.0108	0.0001		
Fungal biomass (ergosterol)	ANOVA	**Leaf species**	2	396.2	198.1	21.118	***p* < 0.001**
		**Fungicide**	4	2751.7	687.9	73.341	***p* < 0.001**
		**Leaf species** **×** **fungicide**	8	290.5	36.3	3.872	***p* < 0.001**
		Residuals	135	1266.3	9.4		
Bacterial density	ANOVA	**Leaf species**	2	1.25 × 10^18^	6.26 × 10^17^	31.205	***p* < 0.001**
		**Fungicide**	4	2.10 × 10^17^	5.25 × 10^16^	2.618	**0.038**
		Leaf species × fungicide	8	1.37 × 10^17^	1.71 × 10^16^	0.855	0.557
		Residuals	130	2.61 × 10^18^	2.01 × 10^16^		

*Df* degrees of freedom, *Sum Sq *sum of squares, *Mean Sq* mean squares

*p*-values printed bold indicate statistical significance

In this study, alder was decomposed faster than maple and beech despite lower levels of alder-associated fungal biomass (Fig. 1; Table 2). Fungal biomass ignores the AH (aquatic hyphomycete) species composition and the potential replacement of less active fungal species by species with a higher decomposition efficiency (Baudy et al. 2021). Moreover, the alder-associated fungal biomass might have already peaked before the termination of the experiment (Baldy et al. 1995). This assumption is supported by Artigas et al. (2004), who reported a peak in alder-associated ergosterol levels after 14 days under optimal conditions. Contrarily, for maple and beech, the maximum of ergosterol may not have been reached at test termination.

**Table 2:** Bacterial density, as number of cells per mg leaf dry weight, and ergosterol concentration, as µg per mg of leaf dry weight, of different leaf species (alder, maple, and beech) ± 95% CIs., for the increasing fungicide concentrations

Leaf species	Fungicide concentration (µg/L)	Bacterial density (number of cells 10^8^/mg leaf dw)	Ergosterol concentration (µg/mg leaf dw)
Alder	0	3.04 ± 0.68	8.40 ± 1.17
	3	3.33 ± 0.44	6.55 ± 1.07
	30	2.08 ± 0.21	6.90 ± 1.10
	300	2.48 ± 0.40	4.86 ± 0.92
	3000	2.40 ± 0.29	0.56 ± 0.15
Maple	0	3.49 ± 0.27	14.11 ± 0.80
	3	4.60 ± 0.79	14.79 ± 1.00
	30	3.90 ± 0.64	11.03 ± 0.99
	300	2.56 ± 0.19	5.90 ± 0.82
	3000	3.52 ± 0.28	0.82 ± 0.06
Beech	0	1.33 ± 0.10	12.70 ± 0.75
	3	1.53 ± 0.24	11.82 ± 1.20
	30	1.67 ± 0.19	11.54 ± 1.03
	300	0.88 ± 0.10	3.87 ± 0.43
	3000	1.51 ± 0.08	0.14 ± 0.04

Fungicide exposure negatively impacted leaf litter decomposition, fungal biomass and partially bacteria density for all leaf species (Fig. 1, Tables 1 and S3; *p* < 0.05). Although the observed effect sizes were small (5%–12%), likely due to the fungicide concentrations not being high enough to impact fungicide-tolerant AH species (Zubrod et al. 2019), leaf litter decomposition rates decreased with increasing fungicide concentrations independent of the leaf species (Fig. 1). The interaction term of the factor “leaf species” and “fungicide” was non-significant (*p* > 0.9; Tables 1 and S3, Fig. S1), which points to a similar response pattern of the microbial communities in terms of leaf litter decomposition among leaf species with increasing fungicide concentrations. Nevertheless, the highest reductions in decomposition rates varied by a factor of two (12 vs. 21% and 20% reduction for alder, maple, and beech, respectively, between control and 3000 μg/L; Table S2) pointing to relevant differences between leaf species. While the reductions between the second highest (i.e., 300) and highest (i.e., 3000 μg/L) treatment were also noteworthy (i.e., 14%, 7% and 34% for alder, maple and beech, respectively). These reductions of leaf decomposition support the negative impact of the fungicide mixture, which tended to increase with less favourable leaf species traits (higher recalcitrance and decreasing nutrient levels) and was particularly pronounced for fungal biomass (Table 2). In contrast to fungal biomass, bacterial density differed slightly between maple and alder but was reduced for beech, independent of the fungicide concentrations. Hence, consistent pattern in bacteria density was not observed, supporting their minor contribution to leaf decomposition (Hieber and Gessner 2002).

For the tested fungicide concentrations, no significant changes in decomposition rates were found for alder in comparison to the control. In a previous study (Zubrod et al. 2015) with the same fungicide mixture at comparable concentrations, however, significant changes in the leaf decomposition rate were detected for alder, which might be related to a substantially higher statistical power due to higher replication (n = 49) relative to the present study (n = 10). Nonetheless, the effect size observed for alder at the highest fungicide concentration (i.e., 3000 μg/L) is in accordance with Zubrod et al. (2015). For the other leaf species, the decomposition rate was affected similarly between maple and beech, with effect size being twice as high when compared to alder. Maple and beech showed a non-significant reduction in the leaf decomposition rate of up to ~ 20% at the two highest fungicide concentrations (300–3000 μg/L). Changes in fungal biomass support this pattern (see also Zubrod et al. 2015), with a lower reduction of the ergosterol concentration on alder relative to beech or maple among fungicide treatments (Table 2). Moreover, fungal biomass was the only evaluated endpoint to show an interaction between leaf species and fungicide exposure, suggesting a non-additive effect of both variables. Based on our within species data, the latter findings suggest that traits of alder leaves (high nutrient levels and low recalcitrance) enable leaf-associated microorganisms to acquire leaf-bound energy more easily to withstand potential effects induced by fungicide exposure (Solé et al. 2012). This interpretation has not been supported by statistical significance (Table 1), however it is backed by fungal biomass data being more reduced under fungicide exposure on the most recalcitrant and least nutrient-rich leaf species (namely beech)—an observation made by Artigas et al. (2004). In their study, the presence of 30 μg tebuconazole/L induced a 60% higher reduction in fungal biomass associated with more recalcitrant black poplar (*Populus nigra* L.) relative to alder. The discrepancies in fungicide effects between maple and alder, which both should be comparably well decomposable, might be related to maple having a relatively smooth surface on both leaf sides making colonisation and penetration by fungi more challenging (Kearns and Bärlocher 2008). Consequently, fungal propagules are exposed to fungicides for a longer duration. On alder, however, the fungal propagules can quickly attach and grow into the leaf (Kearns and Bärlocher 2008), which may provide protection and reduced fungicide exposure. Moreover, some fungicides only act on the propagules of fungi and not on growing mycelium (Escudero-Leyva et al. 2022). While this aspect seems of little relevance in absence or at low levels of fungicides, the combination of leaf surface traits with fungicide stress may have contributed to the more pronounced fungicide effect at higher concentrations in beech and maple leaves. Similarly, bacterial density was not substantially affected by fungicide exposure (Table S3), suggesting again a minor relevance of leaf recalcitrance and nutrient content for bacterial colonisation (Feckler et al. 2017).

## Conclusion

Overall, this study shows that higher recalcitrance and lower nutrient levels in leaf litter potentially may lead to increased fungicide effects during its decomposition. This seems particularly relevant in the light of alder replacement in riparian zones over the last decades across Europe due to different causes, such as habitat exploitation and pathogen infections (Brasier et al. 1995, 1999, 2004; Graça and Canhoto 2006; Richardson et al. 2007; Husson et al. 2015). Therefore, changes in tree species composition along riverbanks are expected (Bjelke et al. 2016) further diversifying the leaf litter and its susceptibility to be decomposed. Thus, understanding the leaf litter decomposition activity of local microbial communities is essential to expand our research on how leaf litter traits interact with the impact of chemical stressors.

## Supplementary Information

Below is the link to the electronic supplementary material.

Supplementary file1 (PDF 235 kb)
