# Supplementary material for: Leaf Species-Dependent Fungicide Effects on the Function and Abundance of Associated Microbial Communities
Source: Bull Environ Contam Toxicol. 2023 May 9;110(5):92. doi: 10.1007/s00128-023-03728-2 (PMC10169882; doi:10.1007/s00128-023-03728-2)
Supplement: Supplementary file 1 — Supplementary file1 (PDF 235 kb) [file 128_2023_3728_MOESM1_ESM.pdf]

Supplementary information for the paper:

Leaf species-dependent fungicide effects on the function and abundance of associated microbial communities

Sara Gonçalves[1], Ruben Post[1], Marco Konschak[1], Jochen Zubrod[1,2], Alexander Feckler[1,3], Mirco Bundschuh [1,4*]

[1]iES Landau, RPTU Kaiserslautern-Landau, Fortstrasse 7, 76829 Landau, Germany
[2]Zubrod Environmental Data Science, Friesenstrasse 20, 76829 Landau, Germany
[3]Eußerthal Ecosystem Research Station, RPTU Kaiserslautern-Landau, Birkenthalstraße 13, 76857 Eußerthal, Germany
[4]Department of Aquatic Sciences and Assessment, Swedish University of Agricultural Sciences, Box 7050, 750 07 Uppsala, Sweden
Corresponding author*: mirco.bundschuh@rptu.de

Table S1. Information on the fungicide mixture components, their product names, manufacturers, active ingredient concentrations, nominal concentrations, and mode of action.

Substance	Product name	Manufacturer	Active ingredient concentration	Nominal concentrations (µg/L)	Mode of action according to the Fungicide Resistance Action Committee (2017)
Azoxystrobin	Ortiva	Syngenta Agro	250 g/L	0; 0,5; 5; 50; 500	Inhibition of mitochondrial respiration
Carbendazim	Derosol	Bayer Crop Science	600 g/kg	0; 0,5; 5; 50; 500	Inhibition of mitosis and cell division
Cyprodinil	Chorus	Syngenta Agro	500 g/kg	0; 0,5; 5; 50; 500	Inhibition of amino acid and protein synthesis
Quinoxyfen	Fortess 250	Dow Agro Science	250 g/L	0; 1; 10; 100; 1000	Perturbation of signal transduction
Tebuconazol	Folicur	Bayer Crop Science	250 g/L	0; 0,5; 5; 50; 500	Inhibition of sterol biosynthesis

Table S2. **L**eaf litter decomposition rate, k, per day, of increasing total fungicide concentrations for the different leaf species alder, maple, and beech.

**Leaf species**	**Fungicide concentration (µg/L)**	**Leaf litter decomposition rate ± sd**		
Alder	0	0.034	±	0.013
	3	0.032	±	0.014
	30	0.029	±	0.011
	300	0.030	±	0.005
	3000	0.026	±	0.011
Maple	0	0.031	±	0.005
	3	0.034	±	0.006
	30	0.031	±	0.010
	300	0.026	±	0.007
	3000	0.025	±	0.008
Beech	0	0.013	±	0.006
	3	0.012	±	0.005
	30	0.015	±	0.007
	300	0.012	±	0.012
	3000	0.008	±	0.007

Table S3. Statistical output of pairwise comparisons between the individual fungicide concentrations using Wilcoxon rank sum tests with subsequent Bonferroni correction. p-values printed bold indicate statistical significance.

Endpoint	Comparision	alder	beech	maple
Leaf litter decomposition rate	0-3	1	1	1
	0-30	1	1	1
	0-300	1	1	1
	0-3000	1	0.4	0.63
	3-30	1	1	1
	3-300	1	1	0.185
	3-3000	1	1	**0.052**
	30-300	1	1	1
	30-3000	1	0.45	1
	300-3000	1	1	1
Fungal biomass (ergosterol)	0-3	1	1	1
	0-30	1	1	0.23231
	0-300	0.3428	**0.0001**	**0.00011**
	0-3000	**0.0018**	**0.00163**	**0.00011**
	3-30	1	1	0.28806
	3-300	1	**0.01505**	**0.00022**
	3-3000	**0.0027**	**0.00163**	**0.00011**
	30-300	1	**0.00022**	0.0105
	30-3000	**0.0061**	**0.00163**	**0.00011**
	300-3000	**0.0044**	**0.00163**	**0.00487**
Bacterial density	0-3	1	1	1
	0-30	1	1	1
	0-300	1	0.147	0.29
	0-3000	1	1	1
	3-30	0.74	1	1
	3-300	1	0.63	0.75
	3-3000	1	1	1
	30-300	1	0.068	0.35
	30-3000	1	1	1
	300-3000	1	**0.015**	0.19

Table S4. Fitted models and their respective parameterization separated by leaf species.

Leaf species	Model	Lower limit	Parameters	
alder	Weibull type (3 parameters)	0	b:	0.532
			c:	0.033
			e:	38965.000
maple	Log-logistic (log(ED50) as parameter)	0	b:	0.351
			c:	0.032
			e:	11.423
beech	Weibull type (3 parameters)	0	b:	0.712
			c:	0.014
			e:	6372.800

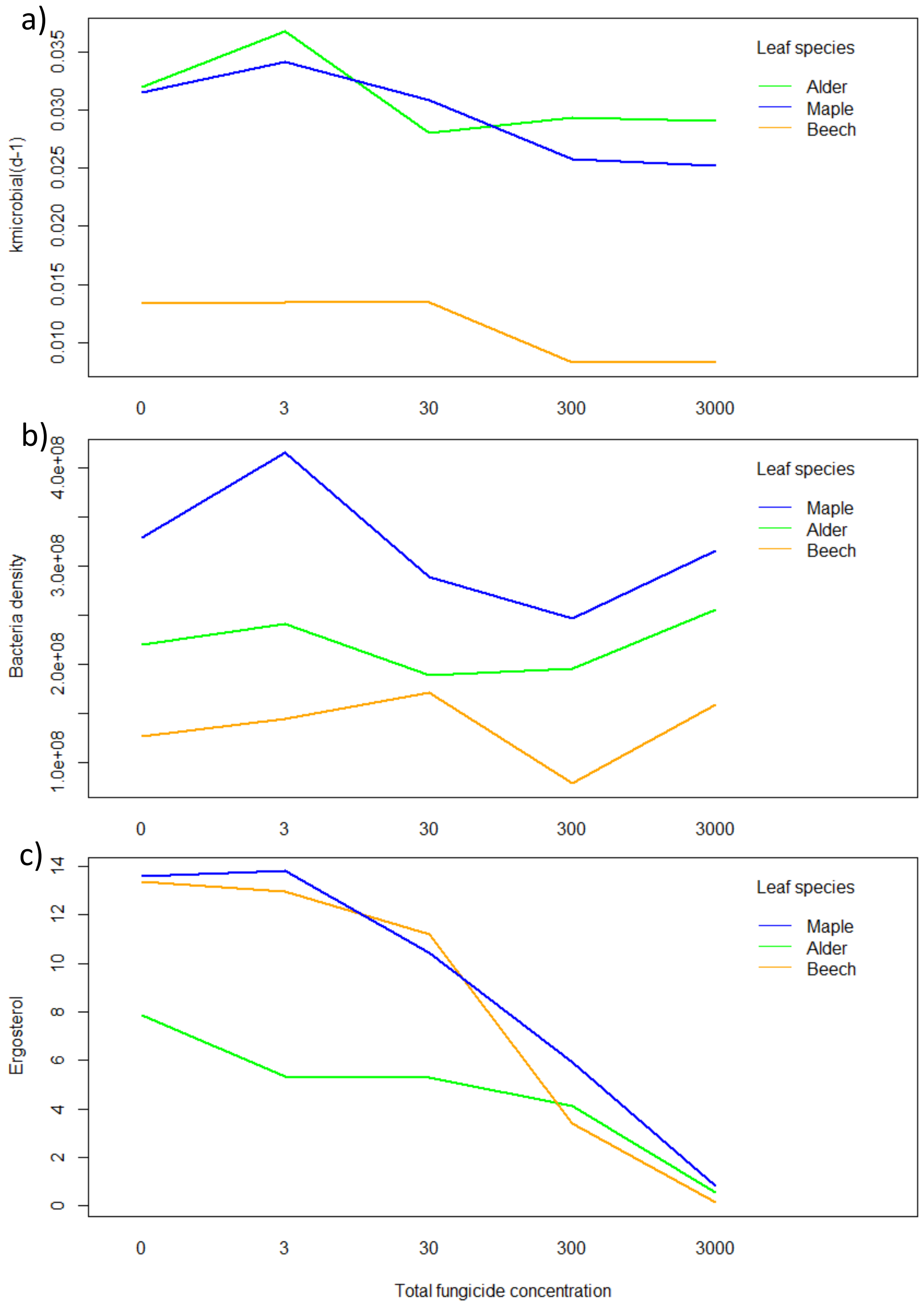

Figure S1- Interactions plots between factors "Fungicide" and "Leaf species" for a) Leaf decomposition rate; b) Bacterial density; and c) Ergosterol (Fungal biomass estimate). Lines in green, blue and orange indicate different leaf species, Alder, Maple and Beech, respectively. If the two lines on the interaction plot are parallel, then there is no interaction effect. If the lines intersect, then there is likely an interaction effect.
